# Effect of paternal age on intracytoplasmic sperm injection outcomes in cryptozoospermic men: Ejaculated or testicular sperm?

**DOI:** 10.1097/MD.0000000000016209

**Published:** 2019-06-28

**Authors:** Yang Yu, Ruixue Wang, Qi Xi, Hongguo Zhang, Yuting Jiang, Leilei Li, Ruizhi Liu, Xinyue Zhang

**Affiliations:** Center for Reproductive Medicine, Center for Prenatal Diagnosis, First Hospital, Jilin University, Changchun, China.

**Keywords:** cryptozoospermia, ejaculated sperm, ICSI outcomes, paternal age, testicular sperm

## Abstract

It is not clear whether age has any influence on the outcomes for sperm used for assisted reproductive technology in cryptozoospermic men. We evaluated intracytoplasmic sperm injection (ICSI) outcomes using ejaculated or testicular sperm in men with cryptozoospermia from different paternal age ranges.

We conducted a retrospective observational study of 35 men with cryptozoospermia who underwent ICSI from 2010 to 2018. They were classified into 2 groups based on male age, namely < 35 years and ≥ 35 years. Each group was further divided into 2 subgroups according to the origin of sperm (ejaculated or testicular).

In the <35 years group, the normal fertilization and high-quality embryo rates for ejaculated sperm were significantly higher than with testicular sperm (74.7% vs. 62.4%, *P* = .02; 50.5% vs. 36.6%, *P* = .03, respectively). However, in the ≥35 years group, the high-quality embryo and clinical pregnancy rates were significantly lower in the ejaculated sperm subgroup than in the testicular sperm subgroup (26.2% vs. 63%, *P* = .002; 12.5% vs. 71.4%, *P* = .04, respectively).

This study indicates that ICSI should be performed as soon as possible for men with cryptozoospermia. When the paternal age ≥35 years, testicular sperm should be used for ICSI, as this offers better high-quality embryo and clinical pregnancy rates.

## 1 Introduction

Cryptozoospermia is defined by the World Health Organization (WHO) as sperm being absent from a fresh semen sample but still observed after centrifugation.^[1]^ Intracytoplasmic sperm injection (ICSI) has been used successfully for men with severe spermatogenic failure, including cryptozoospermia.^[2]^ Sometimes sperm are not found in the ejaculates from men with cryptozoospermia even after centrifugation, which is considered virtual azoospermia.^[3]^ When no sperm can be found in ejaculates on the day of oocyte retrieval, it might be possible to consider testicular sperm extraction. One central concern is whether the origin of spermatozoa (ejaculated or testicular) has any influence on ICSI outcomes for couples requiring assisted reproductive technology (ART) to conceive.

Few studies have compared the outcomes of ICSI between testicular or ejaculated sperm in cases of cryptozoospermia or virtual azoospermia, and the data in the literature are controversial.^[4–11]^ Some investigations reported that the ICSI outcomes using ejaculated sperm for such men were better than with testicular sperm.^[5]^ Others showed contrary conclusions.^[6–10]^ Still others suggested that there was no difference between these sources.^[4,11]^ However, no study has examined the effect of paternal age on ICSI outcomes for men with cryptozoospermia. With increased male age, semen quality will decrease in men with cryptozoospermia, and sometimes it might even develop into nonobstructive azoospermia (NOA). Sperm suffer oxidative stress and sperm DNA fragmentations increase during the process of sperm transport through and storage in the male genital tract.^[12]^ Because of the increased DNA fragmentation rate with the advancing paternal age, concerns of a decreased pregnancy rate with ejaculated sperm are pertinent. To date, the ICSI outcomes in older men with cryptozoospermia after using ejaculated or testicular sperm are not well documented.

Therefore, here we compared our results for ICSI using ejaculated or testicular sperm for men with cryptozoospermia from 2 different age ranges. The aim of the present study is to assess whether the origin of the sperm has an impact on ICSI outcomes at different paternal ages.

## 2 Materials and methods

This was a retrospective observational study that received institutional review board approval from the Medical Ethics Committee of First Hospital of Jilin University (2010-084) and written informed consent was obtained from all patients. The data used to support the findings of this study are available from the corresponding author upon request.

### 2.1 Patients

We conducted a retrospective study of all infertile men with cryptozoospermia who underwent ICSI in the Reproductive Medicine Center of the First Hospital of Jilin University. From 2010 to 2018, 35 such men were included in our study. Within this group, ejaculated sperm were used in 19 oocyte retrieval cycles (18 patients), 17 patients underwent 1 cycle and 1 patient underwent 2 cycles using ejaculated sperm at the same age. Testicular sperm extracted by testicular sperm aspiration (TESA) or microdissection testicular sperm extraction (micro-TESE) were used in 19 oocyte retrieval cycles (17 patients), 15 patients underwent 1 cycle, and 2 patients underwent 2 cycles but 1 TESE surgery. The second cycle, they used the frozen testicular sperm from the first TESE surgery. We calculated the age of patients when they underwent the TESE surgery. They were classified into 2 groups based on male age, namely < 35 years and ≥ 35 years. Each group was divided into 2 subgroups according to origin (ejaculated or testicular sperm). All patients had been diagnosed with cryptozoospermia using at least 2 different centrifuged semen analyses according to WHO criteria. All patients had sufficient sperm for ICSI. Preoperative factors, including age, the levels of follicle-stimulating hormone (FSH), luteinizing hormone (LH), and testosterone, any presence of varicocele, and any history of testicular cancer or cryptorchidism were analyzed. Testicular volume was measured by ultrasound. The average volume of both testes was used for analysis. All samples were subjected to karyotyping and Y chromosome microdeletion analysis. Three patients had AZFc microdeletions. All the procedures were performed by the same surgeon. Exclusion criteria were as follows: infertility caused by female factors, or a wife with karyotype abnormalities.

### 2.2 Semen processing

Semen samples were collected by masturbation after 48 hours to 7 days of abstinence, and allowed to liquefy for at least 20 minutes at 37°C before analysis. When sperm were not identified on initial analysis, centrifugation at 1500 to 3000 rpm was performed. Subsequently, sperm were counted using the surface of the chamber, and viable motile sperm were found for ICSI.

### 2.3 TESA procedure

Testicular sperm aspiration was performed under local anesthesia with 2% lidocaine. A 21-G needle attached to a 20-mL syringe was inserted through the scrotal skin into the testicle. Negative pressure was created by pulling the syringe plunger while the tip of the needle was moved gently in and out, and the needle was then pulled up slowly. The testicular tissue was placed in culture dishes for immediate microscopic examination.

### 2.4 Micro-TESE procedure

The procedure of micro-TESE has been described previously.^[13]^ Briefly, with the patient under general anesthesia, a midline scrotal incision was made and the tunica vaginalis was opened to expose the tunica albuginea. An equatorial incision was made over the tunica albuginea using a surgical microscope, taking care to avoid vasculature injury. Microdissection was then performed to identify larger and more opaque seminiferous tubules, which were considered more likely to contain sperm. The specimens were immediately examined by an embryologist in the operating room. If no sperm were observed intraoperatively, the testicular tissue was thoroughly examined for the presence of sperm by another embryologist in the embryology laboratory.

### 2.5 Ovarian stimulation and ICSI procedure

Ovarian stimulation, oocyte collection, sperm injection, embryo culture, and embryo transfer were performed as described in our previous article.^[14]^ Along luteal down-regulation protocol was used in the patient's female partner. Gonadotrophin releasing hormone agonist (Tryptorelin, Ferring, Germany) administration was administered in the mid luteal phase of the previous cycle. Ovarian stimulation was started by the administration of recombinant follicle-stimulating hormone (FSH) (Gonal-F; Merck Serono, Geneva, Switzerland) when the was follicular diameter was <5 mm. Ovulation was triggered with a single dose of recombinant human chorionic gonadotropin (Ovidrel, EMD Serono, Switzerland) when 2 or more ovarian follicles reached 18 mm or more in diameter. Oocytes were retrieved 36 to 38 hours after the administration of rhCG.

Retrieved oocytes were incubated in Quinn-1020 (SAGE, Inc, Trumbull) medium supplemented with 5% human serum albumin (SAGE, Inc, USA). Selected sperm were washed into the PVP droplet to remove surrounding debris particles, which could be deleterious for oocyte and resulted embryo. Metaphase II oocytes were injected with normal morphology, and whenever possible, motile sperm for ICSI. ICSI was performed under warmed-stage microscope (Olympus, Tokyo, Japan) at 200× magnification using a Hafman optic system, equipped with hydraulic micromanipulation (Eppendorf, Hamburg, Germany). Oocytes were assessed to determine whether fertilization had occurred at 17 to 19 hours after ICSI. Fertilization was considered to be normal if 2 pronuclei (PN) and 2 polar bodies were identified. The fertilization rate was calculated as the percentage of metaphase II oocytes forming 2 PN. After 72 hours fertilization, according to modified PETER cleavage stage embryos scoring system based on the blastomeric number and symmetry and cytoplasm fragmentation to assess the day 3 embryo quality.^[15]^ Two embryos were transferred into the female partner's uterine cavity on day 3 after oocyte retrieval.

Serum hCG concentrations were measured 14 days after embryo transfer. Clinical pregnancy was defined as the presence of a gestational sac confirmed by ultrasound examination at the fourth week after embryo transfer. Live birth rate was defined as babies/embryo transfer.

### 2.6 Statistical analysis

All statistical data were analyzed with SPSS, version 17.0 (SPSS Inc). For quantitative data such as testis volume, age and hormone levels, independent-sample *t* test was performed to compare. The qualitative variables such as fertilization rate and live birth rate were evaluated by the *χ*^2^ or Fisher exact tests. *P* <.05 was considered statistically significant.

## 3 Results

Baseline characteristics of the study groups are presented in Table 1. There were no significant differences in terms of male body mass index (BMI), testicular volume, or FSH, LH, and testosterone levels (all *P* ≥ .05). The ICSI outcomes of different age groups are shown in Table 2. Thirty-five patients underwent 38 oocyte retrieval cycles. The estradiol levels on the day of hCG administration and the rate of formation of 2-pronuclear zygotes were significantly higher in the < 35 years group than in the ≥ 35 years group (*P* = .002 and *P* = .03, respectively). The implantation, clinical pregnancy, and live birth rates tend to be higher in the < 35 years group, but these differences were not statistically significant.

**Table 1:**
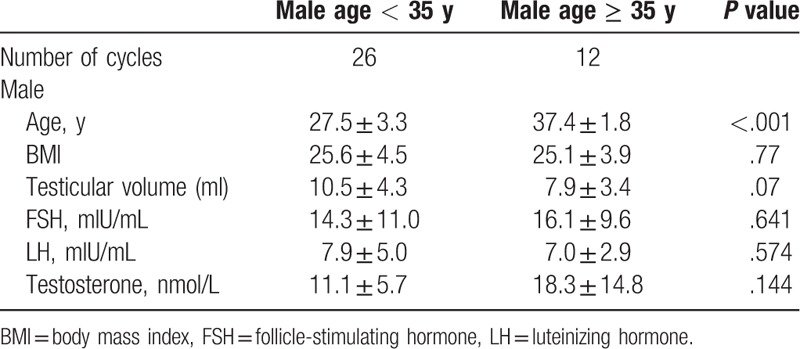
Baseline characteristics of the study groups.

**Table 2:**
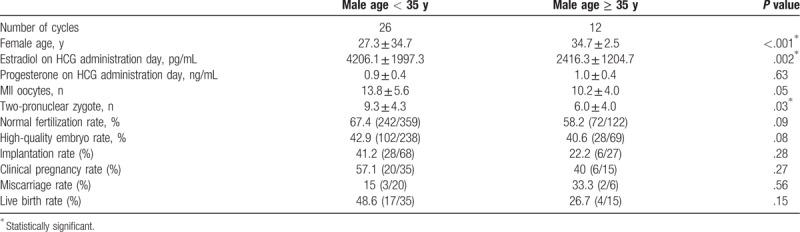
ICSI outcomes of different age groups in men with cryptozoospermia.

ICSI outcomes for the ejaculated sperm and testicular sperm subgroups in different age groups are shown in Table 3. In the < 35 years group, the normal fertilization rate and high-quality embryo rate in men using ejaculated sperm were significantly higher, compared with the testicular sperm subgroup (74.7% vs. 62.4%, *P* = .02; 50.5% vs. 36.6%, *P* = .03, respectively), whereas the implantation, clinical pregnancy, miscarriage, and live birth rates did not differ between them. In the ≥ 35 years group, the mean male age was significantly lower in the ejaculated sperm subgroup (36.3 ± 1.5 vs. 38.5 ± 1.5, *P* = .03), and the high quality embryo and clinical pregnancy rates were significantly lower in the ejaculated sperm subgroup than in the testicular sperm subgroup (26.2% vs. 63%, *P* = .002; 12.5% vs. 71.4%, *P* = .04, respectively). The implantation, miscarriage, and live birth rates showed no significant differences between these subgroups.

**Table 3:**
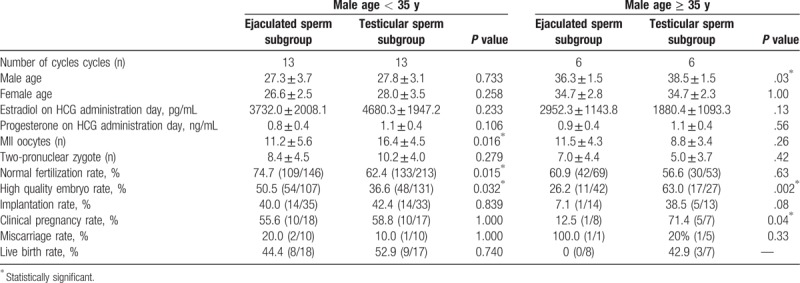
ICSI outcome of ejaculated sperm group versus testicular sperm group in 2 different age groups.

## 4 Discussion

Intracytoplasmic sperm injection is an effective technique for patients with extreme spermatogenic failure. Combined with testicular sperm retrieval technique, ICSI enables patients with increasing rates of cryptozoospermia the opportunity to become biological fathers when multiple attempts at ICSI have failed using ejaculated sperm.^[4,16]^ When no sperm can be found in ejaculates, TESA or micro-TESE are necessary to obtain sufficient viable spermatozoa for ICSI.

The choice between using ejaculated or testicular sperm for ICSI in men with cryptozoospermia will not only help us predict patients’ outcomes but it can also provide better counseling for them. Controversial data have appeared in the literature.^[5–11]^ Thus, Amirjannati et al^[5]^ showed similar fertilization rates between 2 such groups, with regard to using testicular sperm as an invasive surgical procedure, and suggested that the use of ejaculated sperm should be recommended. Other studies suggested that testicular sperm were superior to ejaculated sperm in achieving pregnancies for men with cryptozoospermia. The fertilization rate using testicular sperm was significantly higher, compared with ejaculated sperm.^[6,7,9]^ Moreover, Ben-Ami et al and Cui et al showed that the implantation, clinical pregnancy, and live birth rates were all significantly higher using testicular sperm than with the ejaculated sperm.^[8,10]^ However, a comprehensive meta-analysis by Abhyankar et al^[11]^ showed there was no statistically significant difference in fertilization or pregnancy rates using testicular versus ejaculated sperm from men with cryptozoospermia. One possible reason was that most studies had no further identification in paternal age. Only a few studies have mentioned the paternal age, but no comprehensive research has been done. Abhyankar et al found that a testicular sperm group had a significantly more advanced paternal age than an ejaculated sperm group.^[11]^ Other studies also showed that both paternal and maternal ages were greater in testicular sperm groups.^[7,8]^ The relatively advanced age of couples requiring TESE might be the rationale for using such spermatozoa for ICSI after failure to achieve pregnancy with ejaculated spermatozoa.

A retrospective study by Levitas et al^[17]^ examining semen samples from 6022 men found that peak semen parameters occurred between the ages of 30 to 35 years, and a statistically significant and inverse relationship was observed between sperm quality and patient age. A study by Ford et al involving 585 couples also showed an increased risk of infertility in couples where the male partner was >35 years old, compared with couples among whom the men was < 35 years old.^[18]^ Moreover, Singh et al showed that the percentage of sperm with highly damaged DNA and DNA break number was statistically significantly higher in men aged > 35 years than in those aged < 35 years.^[19]^ In our study, paternal age also had a negative impact on ICSI outcomes. The implantation, clinical pregnancy, and the live birth rates were all higher in the <35 years group than in the ≥35 years group, regardless of the origin of the sperm. The significantly lower estradiol levels and 2PN zygote formation rate in the ≥35 years group might indicate decreased ovarian function in the female partner. In the <35 years group, the normal fertilization and high-quality embryo rates in men with ejaculated sperm subgroup were significantly higher than in the testicular sperm subgroup, whereas the high-quality embryo and clinical pregnancy rates were significantly lower in the ejaculated sperm subgroup than in the testicular sperm subgroup for men aged ≥35 years. The possible reasons involved 2 aspects: First, semen quality is reduced with increased paternal age and prolonged infertility.^[20]^ There is no definite evidence to demonstrate a negative effect of poor semen quality on ART outcomes because successful ICSI requires only a few active sperm and normal sperm morphology,^[21]^ but it is difficult to select satisfactory or even sufficient ejaculated sperm for ICSI from older men with cryptozoospermia. In some cases, cryptozoospermia may develop into NOA, and sufficient sperm might not be retrieved from some patients even using micro-TESE. Second, sperm suffer oxidative stress and nuclear DNA damage during transit through the male genital tract.^[22–24]^ Sperm in men with advanced paternal age are more vulnerable to such damage. Increased DNA fragmentation indices have been associated with worse pregnancy outcomes.^[19]^ Therefore, ICSI should be performed as soon as possible for patients with cryptozoospermia and when the patients are ≥35 years old, it is feasible to use testicular sperm for ICSI.

One limitation of this study was the low number of cases examined. Although the study population was small and the results need further verification, it still has some guiding value for urologists. Future studies should focus on expanding the sample size and the degree of influence on sperm from men of different ages. However, the results of our study may assist both physicians and patients in choosing more rational treatments to achieve pregnancy by ART.

In conclusion, this study indicates that ICSI should be performed as soon as possible for men with cryptozoospermia. When the paternal age <35 years, the use of ejaculated sperm can achieve a higher live birth rate. When the paternal age is ≥35 years, testicular sperm should be recommended for ICSI in men with cryptozoospermia, as it offers better high-quality embryo and clinical pregnancy rates.

## Acknowledgments

Thanks to the statistical consultation provided by the Epidemiology Research Center of the First Hospital of Jilin University.

## Author contributions

Authorship: YY is the first author, collected the sample, and wrote the article; RW was responsible for data analysis and statistical analysis; QX and YJ performed the sample analysis; HZ and LL collected the cases; RL performed critical revision of article; XZ involved in the critical revision of the article and final approval of article.

**Conceptualization:** Yang Yu.

**Data curation:** Yang Yu, Ruixue Wang, Qi Xi.

**Formal analysis:** Ruixue Wang.

**Funding acquisition:** Ruizhi Liu.

**Investigation:** Qi Xi, Hongguo Zhang.

**Methodology:** Hongguo Zhang.

**Project administration:** Ruizhi Liu, Xinyue Zhang.

**Resources:** Yuting Jiang.

**Supervision:** Leilei Li, Xinyue Zhang.

**Validation:** Qi Xi, Xinyue Zhang.

**Writing – original draft:** Yang Yu.

**Writing – review & editing:** Ruixue Wang.
